# The Cholera in London

**Published:** 1854-12

**Authors:** 


*The Cholera in London.*—We abstract from the Boston Medical Journal the following account of cholera in London, since September 23d:

| | | | |
|---|---|---|---|
| Week ending | Sept. 30th, | . . . . | 754 |
| " " | Oct. 7th, | . . . . | 411 |
| " " | Oct. 14th, | . . . . | 249 |
| " " | Oct. 21st, | . . . . | 163 |

The total number of deaths during the last named week was 1321, which was 300 above the average for the last ten years, allowing for increase in population.

Vessels from Europe have recently arrived at New York, which lost many passengers by cholera upon the voyage. A medical friend, of much talent, once told us of his experience in a passenger ship, from Liverpool to New York, with cholera on board. He got out of medicine, and at last was reduced to quinine and commercial sulphuric acid, which happened to be in the cargo. With those he succeeded in making a very good average of cures. H.

---

The Medical Society of Virginia offer a medal or some other testimonial of fifty dollars value, for the best essay on Pneumonia.

Communications may be addressed to Dr. D. H. Tucker, at Richmond, Dr. J. S. Davis, of the University of Virginia, or Dr. J. J. Thweatt of Petersburg, at any time before the 1st March, 1855. H.

